# Supplementary material for: Do whispering minds tingle alike? Exploring the relationship between ASMR-sensitivity, trait-ASMR, and trigger preference
Source: PLoS One. 2025 Jul 9;20(7):e0326346. doi: 10.1371/journal.pone.0326346 (PMC12240330; doi:10.1371/journal.pone.0326346)
Supplement: S1 Table — (DOCX) [file pone.0326346.s001.docx]

**S1 Table: Paired samples t-tests by ASMR-15 subscales and by ASMR-sensitivity groups.**

|  | aASMR | nASMR |
| --- | --- | --- |
| AC / Sens | t(15468) = -153.924, p <.001 | t(1209) = -23.635, p <.001 |
| AC / Relax | t(15468) = -251.616, p <.001 | t(1209) = -67.242, p <.001 |
| AC / Affect | t(15468) = -166.417, p <.001 | t(1209) = -38.825, p <.001 |
| Sens / Relax | t(15468) = -96.533, p <.001 | t(1209) = -36.471, p <.001 |
| Sens / Affect | t(15468) = -2.377, p <.019 | t(1209) = -12.320, p <.001 |
| Relax / Affect | t(15468) = 95.813, p <.001 | t(1209) = 31.033, p <.001 |
