# Supplementary material for: Do whispering minds tingle alike? Exploring the relationship between ASMR-sensitivity, trait-ASMR, and trigger preference
Source: PLoS One. 2025 Jul 9;20(7):e0326346. doi: 10.1371/journal.pone.0326346 (PMC12240330; doi:10.1371/journal.pone.0326346)
Supplement: S2 Table — (DOCX) [file pone.0326346.s002.docx]

**S2 Table: Independent samples t-test by subscales of the ASMR-15 between cluster groups.**

|  | High / Medium | High / Low | Medium / Low |
| --- | --- | --- | --- |
| AC | t(119224.181) = 160.033, p <.001 | t(6373.450) = 94.514, p <.001 | t(6827.898) = -14.734, p <.001 |
| Sens | t(12486.091) = 1.280, p =.193 | t(6753.272) = 85.630, p <.001 | t(6547.031) = 85.174, p <.001 |
| Relax | t(10208.077) = 15.237, p <.001 | t(4935.665) = 32.218, p <.001 | t(5876.047) = 22.707, p <.001 |
| Affect | t(11311.311) = 28.856, p <.001 | t(6673.217) = 117.696, p <.001 | t(7687.737) = 89.334, p <.001 |
