# Supplementary material for: Do whispering minds tingle alike? Exploring the relationship between ASMR-sensitivity, trait-ASMR, and trigger preference
Source: PLoS One. 2025 Jul 9;20(7):e0326346. doi: 10.1371/journal.pone.0326346 (PMC12240330; doi:10.1371/journal.pone.0326346)
Supplement: S3 Table — (DOCX) [file pone.0326346.s003.docx]

**S3 Table: Paired samples t-tests by ASMR-15 subscales between trait-ASMR cluster groups.**

|  | High | Medium | Low |
| --- | --- | --- | --- |
| AC / Sens | t(7069) = -83.824, p <.001 | t(5542) = -230.516, p <.001 | t(4065) = -50.423, p <.001 |
| AC / Relax | t(7069) = -177.447, p <.001 | t(5542) = -302.072, p <.001 | t(4065) = -131.122, p <.001 |
| AC / Affect | t(7069) = -113.662, p <.001 | t(5542) = -206.596, p <.001 | t(4065) = -52.978, p <.001 |
| Sens / Relax | t(7069) =- 66.292, p <.001 | t(5542) = -47.432, p <.001 | t(4065) = -77.477, p <.001 |
| Sens / Affect | t(7069) = -23.813, p <.001 | t(5542) = 6.901, p <.001 | t(4065) = 5.152, p <.001 |
| Relax / Affect | t(7069) = 46.498, p <.001 | t(5542) = 53.828, p <.001 | t(4065) = 101.182, p <.001 |
