# Supplementary material for: Do whispering minds tingle alike? Exploring the relationship between ASMR-sensitivity, trait-ASMR, and trigger preference
Source: PLoS One. 2025 Jul 9;20(7):e0326346. doi: 10.1371/journal.pone.0326346 (PMC12240330; doi:10.1371/journal.pone.0326346)
Supplement: S4 Table — (DOCX) [file pone.0326346.s004.docx]

**S4 Table: Full Principal Component Analysis**

| Trigger | 1 | 2 | 3 | 4 | 5 | 6 | 7 | 8 | 9 | 10 | 11 | 12 | 13 |
| --- | --- | --- | --- | --- | --- | --- | --- | --- | --- | --- | --- | --- | --- |
| Sales roleplay | .716 | .123 |  | .204 |  |  |  |  |  |  |  |  | .182 |
| Interviewing for job | .696 |  |  |  |  |  |  |  |  |  |  |  |  |
| Customer services roleplay | .688 | .142 | .155 | .207 |  |  |  |  |  |  |  |  | .216 |
| Consultation roleplay | .630 |  | .242 | .292 |  |  |  |  |  |  |  |  | .200 |
| Q&A roleplay | .627 | .119 |  |  |  |  |  | .168 |  | .123 |  |  |  |
| Taking a survey | .626 | .143 | .168 |  |  |  |  |  |  | .140 |  |  | -.130 |
| Pharmacist consultation | .608 | .118 | .390 | .105 |  |  |  |  |  |  |  |  |  |
| Teacher assistance | .533 | .232 | .208 |  |  |  | .127 |  |  |  |  | .156 |  |
| Watching sort items | .150 | .691 |  |  |  | .203 |  |  |  |  |  |  |  |
| Watching perform activity | .129 | .626 |  | .255 |  |  |  |  |  | .100 |  |  |  |
| Watching someone read / browse | .195 | .596 |  |  |  |  |  | .215 |  | .112 |  |  |  |
| Watching someone unwrap | .119 | .589 |  |  |  | .233 |  |  | .151 |  |  |  | .197 |
| Watching someone paint / draw |  | .582 |  | .163 |  | .107 |  |  | -.111 |  | .193 | .153 | -.245 |
| Watching play game | .119 | .487 |  |  |  |  |  |  |  | .444 |  |  | -.118 |
| Towel folding tutorials | .255 | .462 |  |  | .198 |  |  | -.160 | .121 |  |  | .109 | .293 |
| Cranial nerve exam | .158 |  | .801 |  |  |  |  |  |  |  |  |  |  |
| Eye exam | .192 |  | .795 | .111 |  |  |  |  |  |  |  |  |  |
| Medical appointments | .349 |  | .707 | .113 |  |  |  |  |  |  |  |  |  |
| Clinical roleplay | .427 |  | .614 | .237 |  |  |  |  |  |  |  |  |  |
| Face product application |  | .128 | .140 | .758 | .152 |  | .154 |  |  |  |  |  |  |
| Beauty care roleplay | .291 |  | .178 | .715 |  |  |  |  |  |  |  |  | .127 |
| Makeup brushes on face / body |  |  |  | .568 | .321 | .153 | .281 |  |  |  |  |  | -.133 |
| Hairstyling | .107 | .214 |  | .537 |  |  | .454 |  |  |  |  |  |  |
| Receiving personal attention roleplay | .194 |  | .382 | .397 | .159 |  |  | .117 |  |  |  | .113 | .189 |
| Gentle hand movements / swaying |  |  |  |  | .770 | .108 |  |  |  |  | .101 |  | .107 |
| Slow movements |  | .138 |  |  | .744 | .106 |  |  |  |  |  |  | .121 |
| Face touching / tapping |  |  | .163 | .385 | .542 | .135 | .258 | .135 |  |  |  |  | -.133 |
| Tracing on skin with finger |  |  |  | .140 | .491 |  | .471 |  |  |  |  |  | -.138 |
| Someone swaying | .134 |  |  |  | .416 |  |  |  | .113 | .145 | .387 |  |  |
| Hand care | .178 | .217 |  | .259 | .329 |  | .306 |  | .149 |  | .142 |  |  |
| Tapping sounds |  |  |  |  | .215 | .677 |  | .149 |  |  |  |  |  |
| Item sounds |  | .267 |  | .142 |  | .631 |  |  |  |  | .129 |  |  |
| Scratching sounds |  |  | -.107 |  | .138 | .585 | .255 |  |  |  |  |  | -.143 |
| Crinkling sounds |  | .204 |  |  |  | .545 |  |  | .332 |  |  |  | .228 |
| Crisp sounds |  | .140 |  |  |  | .471 | .118 |  | .253 |  | .107 |  | .114 |
| Mechanical sounds | .171 | .304 | .245 | .102 | -.186 | .404 |  |  |  |  | .109 | .173 |  |
| Repetitive sounds |  |  |  |  | .310 | .376 |  | .265 | .112 |  |  | .116 | -.182 |
| Scalp / back massage |  |  |  |  | .164 |  | .798 |  |  |  |  |  |  |
| Hair / scalp touching |  |  |  | .170 |  | .149 | .793 |  |  |  |  |  |  |
| Washing / cutting hair |  |  | .205 | .357 | -.191 | .138 | .540 |  |  |  | .155 |  |  |
| Audible whispering |  |  |  | .108 | .106 |  |  | .660 |  |  |  |  | .319 |
| Rambling | .196 | .187 |  |  |  |  |  | .495 |  |  | .101 | .170 |  |
| Binaural sounds |  | -.171 | .236 |  | .130 | .280 | .117 | .447 | .147 | .100 |  |  |  |
| Reading | .218 | .425 |  |  |  |  |  | .447 |  |  | .247 | .165 |  |
| Inaudible whispering | -.117 |  | .132 |  | .181 |  |  | .437 | .309 |  |  | .141 | -.244 |
| Sticky finger sounds |  | .103 |  |  | .148 | .225 |  |  | .648 |  |  |  |  |
| Squishing sounds |  |  |  |  |  | .161 | .103 |  | .616 |  | .305 |  |  |
| Mouth sounds |  |  |  |  |  |  |  | .228 | .599 |  | -.155 | .146 | -.148 |
| Movie / software sound effects |  |  |  |  |  |  |  |  |  | .636 | .232 |  | .137 |
| Device / video game demos | .152 | .308 |  |  |  |  |  | .104 |  | .591 |  |  |  |
| Magic tricks |  | .103 |  |  |  |  |  |  |  | .558 |  |  |  |
| Rude / demeaning to viewer roleplay | .230 | -.144 |  | .123 |  |  |  |  |  | .393 |  | .134 |  |
| Nature sounds |  |  |  |  |  | .171 | .107 | .154 |  |  | .668 |  |  |
| Coloured lights changing colour |  |  | .127 |  | .232 |  |  |  |  | .198 | .604 |  |  |
| Speaking in an accent | .181 | .117 |  |  |  |  |  |  |  |  |  | .757 | .183 |
| Speaking in a foreign language |  |  |  |  |  |  |  | .188 | .108 |  | .139 | .730 |  |
| Soft speaking | .171 | .125 |  |  |  |  |  | .184 | -.185 |  | .101 | .160 | .551 |
| Being sketched / painted | .213 | .233 | .218 | .333 |  |  | .107 |  | -.127 |  | .173 | .143 | -.352 |
