# Supplementary material for: Do whispering minds tingle alike? Exploring the relationship between ASMR-sensitivity, trait-ASMR, and trigger preference
Source: PLoS One. 2025 Jul 9;20(7):e0326346. doi: 10.1371/journal.pone.0326346 (PMC12240330; doi:10.1371/journal.pone.0326346)
Supplement: S6 Table — (DOCX) [file pone.0326346.s006.docx]

**S6 Table: Inferential statistics for two principal component analyses by trait-ASMR and ASMR-sensitivity groups**

| Factor label | Trait-ASMR | df (2, 16676) | | ASMR- sensitivity | df (1, 16677) | |
| --- | --- | --- | --- | --- | --- | --- |
| PCA01 | F | p | η² | F | p | η² |
| Roleplay | 62.958 | < .001 | .007 | 1.086 | = .179 | .001 |
| Watching | 111.603 | < .001 | .014 | 16.702 | < .001 | .001 |
| IPC | 57.085 | < .001 | .007 | 8.264 | = .004 | .000 |
| Visual | 104.174 | < .001 | .012 | 3.275 | = .070 | .000 |
| Tactile | 122.005 | < .001 | .014 | 38.026 | < .001 | .002 |
| Non-vocal auditory | 36.975 | < .001 | .004 | .162 | = .687 | .000 |
| Vocal auditory | 56.069 | < .001 | .007 | 2.310 | =.129 | .000 |
| Other | 32.177 | < .001 | .004 | 26.586 | < .001 | .002 |
| PCA02 | F | p | η² | F | p | η² |
| Roleplay | 72.710 | < .001 | .009 | 5.182 | = .023 | .000 |
| IPC | 95.048 | < .001 | .011 | 5.342 | = .021 | .000 |
| Tactile | 114.273 | < .001 | .014 | .007 | = .932 | .000 |
| Watching | 36.547 | < .001 | .004 | .016 | = .900 | .000 |
| Non-vocal auditory | 75.374 | < .001 | .009 | .272 | = .602 | .000 |
| Other auditory | 24.775 | < .001 | .003 | 22.377 | < .001 | .001 |
| Other | 52.987 | < .001 | .006 | .646 | = .422 | .000 |
| Vocal auditory | 68.484 | < .001 | .008 | .900 | = .343 | .000 |
