# Supplementary material for: Do whispering minds tingle alike? Exploring the relationship between ASMR-sensitivity, trait-ASMR, and trigger preference
Source: PLoS One. 2025 Jul 9;20(7):e0326346. doi: 10.1371/journal.pone.0326346 (PMC12240330; doi:10.1371/journal.pone.0326346)
Supplement: S7 Table — (DOCX) [file pone.0326346.s007.docx]

**S7 Table: Number and percentage of participant group allocation by ASMR-sensitivity and trait-ASMR**

| Trait-ASMR | Group | n | High | Medium | Low |
| --- | --- | --- | --- | --- | --- |
| ASMR-sensitivity | aASMR | 15469 | 6652 *(43%)* | 5246 *(33.9%)* | 3571 *(23.1%)* |
|  | nASMR | 1210 | 418 *(34.5%)* | 297 *(24.5%)* | 495 *(41%)* |
